# Datasets on the genomic positions of the MLL1 morphemes, the ZFP57 binding site, and ZFBS-Morph overlaps in the build mm9 of the mouse genome

**DOI:** 10.1016/j.dib.2017.05.050

**Published:** 2017-05-28

**Authors:** Minou Bina, Phillip Wyss, Xiaohui C. Song

**Affiliations:** a Purdue University, Department of Chemistry, West Lafayette, IN 47907, USA; b Information Technology at Purdue University, West Lafayette, IN 47907, USA

**Keywords:** CpG-rich motifs, Gene regulation, Genomic imprinting, KMT2A, MLL1 morphemes, Mouse genome, ZFP57 binding site

## Abstract

While MLL1 activates gene expression in most tissues, ZFP57 represses transcription. MLL1 selectively interacts with a group of nonmethylated DNA sequences known as the MLL1 morphemes. ZFP57 associates with a methylated hexamer (ZFBS), dispersed in the genomic DNA segments known as Imprinted Control Regions (ICRs) and germline Differentially Methylated Regions (gDMRs), to maintain allele-specific gene repression. We have identified a set of composite DNA elements (ZFBS-Morph overlaps) that provides the sequence context of ZFBS in the canonical ICRs/gDMRs. This report provides tables listing the nucleotide sequences of the MLL1 morphemes and ZFBS-Morph overlaps. The report also offers links to the data repository at Purdue University, for downloading the positions of the MLL1 morphemes, the ZFP57 binding site, and the ZFBS-Morph overlaps in the mouse genome.

**Specifications Table**

**Table:** 

**Subject area**	Genomics
**More specific subject area**	Gene regulation
**Type of data**	Tables and text files (in bed format, for display at the UCSC genome browser)
**How data was acquired**	Analyzing the mouse chromosomes using Perl Scripts
**Data format**	Tables and text files
**Experimental features**	None
**Data accessibility**	Two links to files deposited at the Purdue University Research Repository:
	1) Bina, M., Wyss, P.J., Wang, D., Song, X.C. (2014). Localization of MLL1 morphemes in mouse mm9 genomic DNA. Purdue University Research Repository. doi:10.4231/R7KW5CXF
	https://purr.purdue.edu/publications/1648/1
	2) Bina, M., Wyss, P.J., Wang, D., Song, X.C. (2014). Localization of MLL1 morphemes in mouse mm9 genomic DNA. Purdue University Research Repository. doi:10.4231/R7KW5CXF
	https://purr.purdue.edu/publications/2473/1

**Value of the data**

• Two tables and three datasets are offered to the scientific community.
• One table lists the nucleotide sequences of the MLL1 morphemes, the other the nucleotide sequences of ZFBS-Morph overlaps.
• Three datasets were created to provide the genomic positions of functionally important DNA sequence-motifs: the MLL1 morphemes, the ZFP57 binding site, and ZFBS-Morph overlaps.
• The datasets consist of two bed files that could be uploaded onto the UCSC genome browser (build mm9 of the mouse genome), to create custom tracks. One file contains the genomic positions of the MLL1 morphemes, the other includes the genomic positions of ZFP57 binding site and ZFBS-Morph overlaps.
• Availability of these datasets facilitates viewing and analyzing genomic positions of functionally important sequence-motifs in the context of the ENCODE data and mapped landmarks including the position of protein-coding genes and CpG Islands.

## 1 Data

**Fig. 1:**
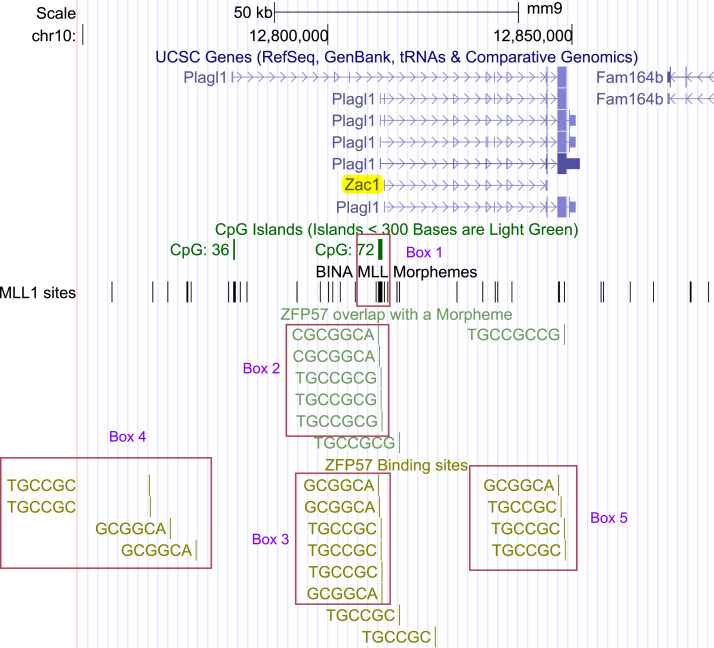
A cluster of ZFBS-Morph overlaps localizes the possible peak position of the *Zac1* gDMR. Box 1 marks the position of CpG72, a conserved CGI that is methylated in oocyte DNA [11]. CpG72 includes a cluster of 5 ZFBS-Morph overlaps, marked by Box 2. As expected, a cluster of ZFBS also is present in CpG72 (Box 3). Random occurrences of ZFBS are marked by Box 4. A cluster of ZFBS also maps to a region that is not part of the gDMR (Box 5). That region includes a single, isolated, ZFBS-Morph overlap. The CGI that is not imprinted (CpG36) does not contain ZFBS-Morph overlaps. The track labeled MLL1 sites shows the position of the MLL1 morphemes in the displayed chromosomal location (chr10:12,749,001–12,879,000). In this relatively long genomic DNA segment (130,000 bps), closely-spaced MLL1 morphemes appear as thick vertical bars, isolated occurrences as thin vertical lines. Clustering of the MLL1 morphemes in CGIs is more apparent in shorter DNA segments; for examples see Refs. [3], [6].

Mixed Lineage Leukemia 1 (MLL or MLL1) is an essential regulator of transcription [1], [2]. MLL1 selectively interacts with a group of nonmethylated DNA sequences known as the MLL1 morphemes: the smallest ‘words’ in DNA that selectively bind the MT-domain in MLL1 [3]. The *MLL1* gene is one of the mammalian orthologs of the Drosophila *Trithorax* [4]. In human cells, functions of MLL1 include gene bookmarking during mitosis, in a manner favoring genes that were highly transcribed during interphase [5]. Gene bookmarking may involve interactions of MLL1 with morphemes that are localized in CGIs: the CpG islands [3]. The MLL1 morphemes contain 2–3 CpGs and occur in both the forward and the reverse orientation in genomic DNA (Table 1). Even though the MLL1 morphemes are dispersed along the chromosomal DNA, often they are clustered in CGIs [3], [6]. Examples include two CGIs (CpG36 and CpG72) associated with the *Plagl1*/*Zac1 loci* (Fig. 1). As a consequence of length-variability of CGIs [7], morpheme-frequencies in the islands vary: for examples, see Refs. [3], [6].

**Table 1:** MLL1 morphemes.

CGACG CGTCG
CGCCG CGGCG
CGCGCG
CGTGCG CGCACG
CGCCCG CGGGCG
CGGACG CGTCCG
CGTACG

In contrast to MLL1, ZFP57 represses transcription [8]. Even though the ZFP57 binding site (ZFBS), a methylated hexamer, is dispersed in many *loci*, the site occurs often in ICRs to maintain allele-specific gene repression [9]. To identify the sequence context of ZFBS in ICRs, we extended the ZFBS length to include a subset of the MLL1 morphemes (Table 2), producing ZFBS-Morph overlaps [10]. Clusters of 2 or more ZFBS-Morph overlaps correctly localized ~90% of the known germline ICRs in the mouse genome [10], Table 3. As an example, Fig. 1 shows a cluster of 5 ZFBS-Morph overlaps in the gDMR of *Zac1*. This cluster is within CpG72, a conserved CGI that is methylated in oocyte DNA [11].

**Table 2:** ZFBS-Morph overlaps.

TGCCGCG	CGCGGCA
TGCCGCCG	CGGCGGCA
TGCCGCGCG	CGCGCGGCA
TGCCGCCCG	CGGGCGGCA
TGCCGCACG	CGTGCGGCA

**Table 3:** Closely-spaced ZFBS-Morph overlaps in the canonical ICRs in the mouse genome. Identical genes that are displayed in 2 rows contain closely-spaced ZFBS-Morph overlaps at two different genomic positions.

**Genomic positions (mm9)**	**Genes**	**ZFBS-Morph overlaps**
chr1:63,246,711-63,246,910	*Gpr1*	TGCCGCCG, CGCGGCA
chr2:157,385,801-157,387,500	*Nnat*	TGCCGCG, CGGGCGGCA, TGCCGCG
chr2:152,512,591-152,512,650	*Mcts2*	TGCCGCG, TGCCGCGCG
chr2:174,121,336-174,121,660	*Gnas*	TGCCGCG, CGCGGCA, TGCCGCG, CGCGCGGCA
chr2:174,124,701-174,125,300	*Gnas*	CGCGGCA, TGCCGCCCG, TGCCGCCCG, TGCCGCCG
chr2:174,152,536-174,154,195	*Gnas_Ex*	TGCCGCCG, CGGCGGCA, TGCCGCCG, TGCCGCCCG
chr2:174,155,591-174,156,025	*Gnas_Ex*	CGGCGGCA, TGCCGCG
chr6:4,697,131-4,698,550	*Peg10*	TGCCGCG, TGCCGCG
chr6:30,687,491-30,688,825	*Mest*	TGCCGCG, CGCGGCA, TGCCGCG, CGGGCGGCA, TGCCGCG, TGCCGCG
chr6:58,856,861-58,857,170	*Nap1l5*	CGCGGCA, CGCGGCA
chr7:67,148,966-67,149,720	*Snrpn*	CGCGGCA, CGCGGCA
chr7:6,681,601-6,683,200	*Peg3*	CGTGCGGCA, CGCGGCA, TGCCGCG, CGCGGCA
chr7:135,831,441-135,832,095	*Inpp5f*	CGCGGCA, TGCCGCG, CGCGGCA, TGCCGCG
chr7:149,765,896-149,766,315	*H19*	CGCGGCA, TGCCGCG, CGCGGCA
chr7:149,767,676-149,767,975	*H19*	TGCCGCCG, CGTGCGGCA, CGCGGCA
chr7:150,481,306-150,481,730	*KvDMR1*	CGCGGCA, TGCCGCG
chr8:125,388,921-125,389,390	*Cdh15*	TGCCGCG, TGCCGCG
chr9:89,774,326-89,775,050	*Rasgrf1*	TGCCGCG, TGCCGCG
chr10:12,810,341-12,811,120	*Zac1*	CGCGGCA, CGCGGCA, TGCCGCG, TGCCGCG, TGCCGCG
chr11:11,925,501-11,926,400	*Grb10*	CGCGGCA, CGCGGCA
chr12:110,764,761-110,766,795	*IG-DMR*	CGCGGCA, CGCGGCA, TGCCGCG, TGCCGCG, TGCCGCG
chr15:72,640,121-72,641,650	*Peg13*	CGCGGCA, CGCGGCA
chr17:12,934,306-12,935,515	*Igf2r*	CGCGGCA, TGCCGCG, CGCGGCA, CGCGGCA, CGCGGCA, TGCCGCG, TGCCGCG

## 2 Methods

We created two text files: one file consisting of the MLL1 morphemes (Table 1), for details see Ref. [3]; the other containing the ZFBS-Morph overlaps (Table 2), for details see Ref. [10]. These two tables include 2 columns displaying complementary pairs of sequences; both pairs are written in 5′ to 3′ direction; a single sequence is shown for complementary pairs with identical sequences. Subsequently, from the UCSC genome browser we downloaded the nucleotide sequences of the build mm9 of the mouse chromosomes [12]. We wrote 2 Perl scripts [3]. We followed the following steps:

• Script 1 opened and read the data in Table 1, to scan the nucleotide sequence of a specified chromosome; the output was a listing of the positions of the MLL1 morphemes along the analyzed chromosome.
• Script 2 read the output of the first script to create a bed file.
• We combined the bed files to obtain the positions of the MLL1 morphemes for the complete set of the mouse chromosomes.
• A ‘header’ was added to the file containing the complete set of the mouse chromosomes.
• The final bed file can be uploaded on the UCSC genome browser to create a custom track for displaying the genomic positions of the MLL1 morphemes along the mouse chromosomes.

The Specifications Table, shown above, provides a link for downloading the file that contains the positions of the MLL1 morphemes in the mouse genome. After you upload the file onto the UCSC genome browser, to create a custom track, the page may display an entire chromosome. You can direct the browser to a specific region by typing in the query box the name of a gene or a desired chromosomal location; for examples see Table 3 and Refs. [13], [14].

Subsequently, we followed a similar approach for obtaining additional bed files for display at the UCSC genome browser. Specifically, we applied a modified form of script 1, using as input a file containing the ZF57 binding site, as a complementary pair of sequences, and the nucleotide sequence of a specified chromosome. Likewise, we applied the modified form of script 1, using as input a file containing the ZFBS-Morph overlaps (Table 2), and the nucleotide sequence of a specified chromosome. The subsequent steps were done as above. The Specifications Table provides a link for downloading the bed file that contains the genomic positions of both ZFBS and the ZFBS-Morph overlaps.

You can upload several datasets to create custom tracks at the UCSC genome browser. At the top of the browser page, use the pull-down menu under ‘view’ to configure the browser to modify the font-size to a larger value; for example see Fig. 1. Under the same menu, you can select PDF to obtain a snapshot for your record or publication.

For data validation, we analyzed results of ChIP assays reporting allele-specific binding of ZFP57 to ICRs/gDMRs [15]. Our approach localized the likely peak-positions of the canonical ICRs/gDMRs in the mouse genome (Table 3); for details see Ref. [10].
