# Supplementary material for: Datasets on the genomic positions of the MLL1 morphemes, the ZFP57 binding site, and ZFBS-Morph overlaps in the build mm9 of the mouse genome
Source: Data Brief. 2017 May 28;13:202–7. doi: 10.1016/j.dib.2017.05.050 (PMC5458072; doi:10.1016/j.dib.2017.05.050)
Supplement: Supplementary file 1 — Supplementary material [file mmc1.pdf]

conflicts of interest: none
